# Treatment with Apocynin Limits the Development of Acute Graft-versus-Host Disease in Mice

**DOI:** 10.1155/2019/9015292

**Published:** 2019-11-03

**Authors:** Barbara Maximino Rezende, Priscila T. T. Bernardes, William Antonio Gonçalves, Carolina Braga de Resende, Rayssa Maciel Athayde, Thiago Vinicius Ávila, Débora Gonzaga Martins, Marina G. M. Castor, Mauro M. Teixeira, Vanessa Pinho

**Affiliations:** ^1^Departamento de Enfermagem Básica, Escola de Enfermagem, Universidade Federal de Minas Gerais, Belo Horizonte, Minas Gerais, Brazil; ^2^Departamento de Morfologia, Instituto de Ciências Biológicas, Universidade Federal de Minas Gerais, Belo Horizonte, Minas Gerais, Brazil; ^3^Hospital das Clínicas, Universidade Federal de Minas Gerais, Belo Horizonte, Minas Gerais, Brazil; ^4^Departamento de Ciências Básicas da Vida, Universidade Federal de Juiz de Fora, Governador Valadares, Brazil; ^5^Departamento de Farmacologia, Instituto de Ciências Biológicas, Universidade Federal de Minas Gerais, Belo Horizonte, Minas Gerais, Brazil; ^6^Departamento de Bioquímica e Imunologia, Instituto de Ciências Biológicas, Universidade Federal de Minas Gerais, Belo Horizonte, Minas Gerais, Brazil

## Abstract

Graft-versus-host disease (GVHD) is the most serious complication limiting the clinical utility of allogeneic hematopoietic stem cell transplantation (HSCT), in which lymphocytes of donors (graft) are activated in response to the host antigen. This disease is associated with increased inflammatory response through the release of inflammatory mediators such as cytokines, chemokines, and reactive oxygen species (ROS). In this study, we have evaluated the role of ROS in GVHD pathogenesis by treatment of recipient mice with apocynin (apo), an inhibitor of intracellular translocation of cytosolic components of NADPH oxidase complex. The pharmacological blockade of NADPH oxidase resulted in prolonged survival and reduced GVHD clinical score. This reduction in GVHD was associated with reduced levels of ROS and TBARS in target organs of GVHD in apocynin-treated mice at the onset of the mortality phase. These results correlated with reduced intestinal and liver injuries and decreased levels of proinflammatory cytokines and chemokines. Mechanistically, pharmacological blockade of the NADPH oxidase was associated with inhibition of recruitment and accumulation of leukocytes in the target organs. Additionally, the chimerism remained unaffected after treatment with apocynin. Our study demonstrates that ROS plays an important role in mediating GVHD, suggesting that strategies aimed at blocking ROS production may be useful as an adjuvant therapy in patients subjected to bone marrow transplantation.

## 1. Introduction

Graft-versus-host disease (GVHD) is an immunological systemic syndrome associated with hematopoietic cell transplantation that is performed to cure many hematological diseases. Recently, it was estimated that about 35-50% of hematopoietic stem cell transplant recipients develop GVHD [1–4]. The degree of inflammation in the gastrointestinal tract, lymphoid organs, lung, and kidney correlates with the severity of the disease and mortality of transplant recipients. Previous studies have demonstrated the role of innate and adaptive immune responses in the development of GVHD and target organ damage [1, 5, 6]. The overproduction of inflammatory mediators and recruitment of effector leukocytes, including macrophages and T cells, causes destruction and loss of function in organs [5, 7, 8]. In addition, GVHD may disrupt the intestinal barrier leading to translocation of bacteria, causing sepsis and multiorgan failure [9, 10]. Many GVHD therapies have been proposed based on immunosuppression, providing increased susceptibility to opportunistic infection and loss of beneficial graft-versus-leukemia effect [2, 10, 11]. Thus, novel therapies are constantly being explored.

It is well known that systemic inflammatory response involves a complex array of proinflammatory molecule production. In this context, our group has demonstrated that the disruption of this cascade by targeting specific mediators might result in the protection against GVHD [12–16]. However, the network of proinflammatory mediators that govern the pathogenesis of GVHD is still an underestimated field, remaining to be explored.

One of the hallmarks associated with inflammatory response is the activation of a powerful oxidative burst, which is implicated in the pathogenesis of a broad range of diseases including cancer, atherosclerosis, and metabolic and infectious diseases [17–20]. The generation of reactive oxygen species (ROS) by cells occurs via several enzymatic systems, but NADPH oxidases and mitochondria are the major sources of intracellular superoxide [21]. ROS are produced by cells that are involved in immune response and can trigger the production of a wide range of proinflammatory molecules. It is also acknowledged that cytokines can lead to increased ROS production [18, 19, 22, 23]. ROS act as both proinflammatory mediators and signaling molecules and have a deleterious action through tissue damage, mainly due to the oxidative modification of structural molecules in the cells [17, 18, 22, 24]. In a previous study, we have shown an increase in ROS in the mouse liver subjected to GVHD. Based on the elimination of GVHD after treatment with fullerol, a nanocomposite with antioxidant properties [13], we hypothesized that ROS seems to be involved in the establishment of GVHD. Despite the evidence of ROS involvement in the pathogenesis of GVHD, description of the effects of GVHD treatment on dinucleotide phosphate oxidase (NOX)-derived ROS production is still lacking.

In order to determine whether overproduction of ROS contributes to the pathogenesis of GVHD in the present study, we tested the impact of apocynin treatment in the prevention of inflammatory response, organ injury, weight loss, and mortality, in mice subjected to GVHD.

## 2. Materials and Methods

### 2.1. Ethics Statement

The animal care and handling procedures were in accordance with the guidelines of the Institutional Animal Care and Use Committee, and the study received prior approval from the Animal Ethics Committee of Universidade Federal de Minas Gerais (protocol number: 191/2012). Animals judged to be moribund were euthanatized with an overdose of anesthesia (100 *μ*l of mixture of ketamine (37.5 mg/ml) and xylazine (2.5 mg/ml), intravenously) and counted as GVHD lethality. At the end of these experimental procedures, the remaining mice were also euthanized with an overdose of anesthetics. In all experiments, the efforts were made to minimize suffering at all times.

### 2.2. Mice

Eight- to 12-week-old C57BL/6 and B6D2F1 (C57BL/6 X DBA/2) were obtained from the Centro de Bioterismo (UFMG) and maintained in our biotery. All mice were housed under standard conditions in a temperature-controlled room (23 ± 1°C) on an automatic 12 h light/dark cycle. The mice had free access to commercial chow and water. The number of mice in each specific group is provided in the figure legend.

### 2.3. Induction of GVHD

#### 2.3.1. Induction of GVHD in B6D2F1 Mice

Recipient B6D2F1 mice were irradiated at 9 Gy total-body radiation (source 60Co) in two doses at 2 h intervals to minimize gastrointestinal toxicity and then given an i.v. infusion of 3 × 10^7^ splenocytes and 1 × 10^7^ BM cells from C57BL/6 donors. The B6D2F1 mice that received splenocytes from B6D2F1 mice (B6D2F1 to B6D2F1) did not develop any disease and were considered the control group.

#### 2.3.2. Induction of GVHD in BALB/c Mice

Recipient BALB/c mice were irradiated with 7 Gy total-body radiation (source 60Co) in two doses at 2 h intervals to minimize gastrointestinal toxicity and then given an i.v. infusion of 1 × 10^7^ splenocytes and 1 × 10^7^ BM cells from C57BL/6 mice. The BALB/c mice that received splenocytes from BALB/c mice (BALB/c to BALB/c) did not develop any disease and were considered the control group. Because of the toxicity of the high level of body irradiation, the recipient mice received an oral suspension of ciprofloxacin (70 mg/l) in their drinking water from 1 d before to 15 d after transplantation. The BM cells and splenocytes were isolated as previously described [13, 25].

### 2.4. Treatment

The apo group was treated with apocynin (3 mg/kg, intraperitoneally) dissolved in sterile PBS-5% ethanol 30 minutes before transplant and each 24 hours until the end of experiments. The vehicle group received PBS-5% ethanol.

### 2.5. Mortality Rate and Assessment of GVHD Clinical Score

After disease induction, mice were monitored daily for survival and evaluated clinically by a standard scoring system that generates a GVHD score comprised of individual scores for weight loss, posture (hunching), activity, fur texture, skin integrity, diarrhea, and occult blood in feces. Following to that, a clinical index was generated by summation of scores of the seven criteria (maximum index = 14), as described previously [16].

### 2.6. Oxidative Stress Analysis

#### 2.6.1. Reactive Oxygen Species (ROS) Production

ROS production was analyzed at the onset of mortality (13 days after transplantation) in bone marrow, spleen, liver, and ileum using 20,70-dichlorodihydrofluorescein diacetate (DCF-DA), as described previously [26, 27]. The isolated cells (10^5^ cells/well) were incubated in 96-well plates with 50 *μ*M of DCF-DA for 30 min at 37° C, and fluorescence was performed in a spectrophotometer (Synergy 2; BioTek, Winooski, VT) with wavelengths of excitation and emission of 480 and 530 nm, respectively.

#### 2.6.2. Lipid Peroxidation

The thiobarbituric acid reactive substance (TBARS, an index of malonyldialdehyde production) levels in the liver and jejunum-ileum were determined according to the method of Ohkawa et al. [28]. Briefly, the liver and jejunum-ileum were homogenized, mixed with trichloroacetic acid, and kept on ice for 30 min to allow protein precipitation, followed by centrifugation. At that point, 30 *μ*l of the clear supernatant was mixed with 270 *μ*l of phosphate buffer 0,1 M (pH 8.5) and 5.5-dithiobis-(2-nitrobenzoic acid) in methanol. The reaction solution absorbance was measured at 415 nm.

### 2.7. Histopathology

A set of experiments was conducted to quantify the histopathological parameters in the intestine and liver, GVHD target organs. Tissue sections were processed for histological analysis as described previously [16, 29] and evaluated by a pathologist. A numerical value was attributed to the changes observed in the intestinal layers (mucosal, lamina propria, muscular, and serosal) and in the liver (degenerative alterations in the parenchyma). Each animal received a score that was generated by summation of all observed changes (maximum index: 9 for intestine and 6 for liver). Histopathological scores were determined for samples that were obtained from mice on day 13 after the transplant, which corresponded to the GVHD mortality phase.

### 2.8. Quantification of Cytokines and Chemokines

The concentrations of cytokines and chemokines were quantified from intestinal or liver homogenates from animals at onset mortality. The tissues were mixed with PBS which contained antiproteases (0.1 mM phenylmethanesulfonylfluoride (PMSF), 0.1 nM benzethonium chloride, 10 mM ethylenediaminetetraacetic acid (EDTA), 20 Kallikrein Inhibitor Units (KIU), and aprotinin A) and 0.05% Tween 20. The samples were centrifuged for 10 min at 10000 rpm and 4°C. Dilutions of the supernatants in PBS and bovine serum albumin (BSA) 0,1% (1 : 3) were immediately analyzed by ELISA. The cytokines and chemokine concentrations were measured according to the manufacturer procedures (R&D Systems, Minneapolis, MN, USA), and the colorimetric reactions were analyzed with a spectrophotometer at a wavelength of 492 nm.

### 2.9. Quantification of Macrophage Infiltration

Macrophages infiltrating in the liver and ileum were indirectly quantified by measuring NAG activity at day 13 after transplant. A portion of 100 mg of the liver or ileum was resuspended in saline 0.9% (4°C) containing 0.15 *v*/*v* Triton X-100 (Merck, Rahway, NJ, USA), homogenized, and centrifuged at 4°C for 10 min at 1500 rpm. The supernatants were collected and assayed immediately for NAG using a 1 : 3 dilution, as described previously [12]. The results were described as relative numbers of macrophages in 100 mg of tissue.

### 2.10. Intravital Microscopy

GVHD was induced, and the mice were treated with apo or vehicle. At day 13 after transplant, the mice were anesthetized, and the intestinal venules were exposed in a perfusion system with warm bicarbonate-buffered saline (pH 7.4). An intravital microscope (ECLIPSE 50i, Nikon, Japan) with a 20-objective lens was used to examine the mesenteric microcirculation. A digital camera (DS-Qi1MC, Nikon, Japan) was used to project the images onto a computer monitor, and the images were recorded for playback analysis with Nikon Imaging Software (Nikon, Kawasaki, Japan). The intestinal venules 40–60 *μ*m were selected, and the numbers of rolling and adherent leukocytes were determined off-line during the video playback analysis. Rolling leukocytes were defined as those cells that moved at a velocity less than that of the erythrocytes within a given vessel. The flux of rolling cells was measured as the number of rolling cells that passed by a given point in the venule per minute. A leukocyte was considered to be adherent if it remained stationary for at least 30 s, and total leukocyte adhesion was quantified as the number of adherent cells in the intravascular space within an area of 100 *μ*m.

### 2.11. Flow Cytometry Analysis

Cells from the spleen, bone marrow, and liver were plated, 1 × 10^6^ cells/well, in a 96-well plate and stained with monoclonal antibodies (1 : 100) to surface markers from T helper lymphocytes (CD3^+^/CD4^+^). The chimera formation was evaluated in the spleen and bone marrow by the stain of H2^b+^/H2^d+^ surface markers. As negative controls were used monoclonal antibodies IgG1 and IgG2a. The cells were incubated with 20 *μ*l/well of antibody solution 30′/4°C, followed by fixation in 4% of paraformaldehyde to further reading in BD FACSCanto™ II.

### 2.12. Statistical Analysis

Data in the text are expressed as the mean ± SEM. Comparisons between the groups were performed by unpaired *t*-test analysis. A log-rank test was used to compare the relevant survival curves. Statistical significance was set as *p* < 0.05, and all graphs and analysis were performed with GraphPad Prism 6 software (GraphPad Software Inc., San Diego, CA, USA).

## 3. Results and Discussion

### 3.1. Treatment with Apocynin Reduced Mortality and Clinical Signs of GVHD

**Figure 1:**
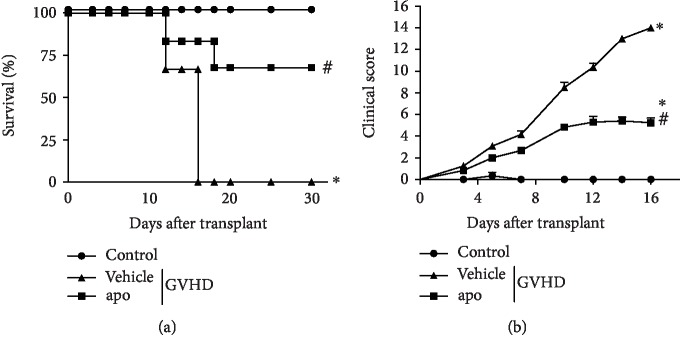
Apocynin treatment is associated with reduced mortality and improvement in GVHD clinical signs. GVHD was induced by the adoptive transfer of 10^7^ BM cells + 3 × 10^7^ splenocytes from C57BL/6 mouse donors to B6D2F1 mice. Mice that received syngeneic (B6D2F1) BM cells and splenocytes did not develop disease and were considered the control group. After GVHD induction, recipient mice were treated with apocynin (3 mg/kg, 24 h/24 h, intraperitoneally) or vehicle 30 min before transplantation until the experimental endpoint. The mice were evaluated every 2 d for survival (a) and clinical scoring (b). The results are shown as means ± SEM, and the numbers of animals were as follows: control group (●, *n* = 5), vehicle group (▲, *n* = 6), and apo group (■, *n* = 6). ∗ and #: *p* < 0.05 compared with the control and vehicle groups, respectively.

We first assessed whether apocynin treatment could prevent GVHD-associated mortality and morbidity. The control group did not develop GVHD, and all mice were alive at the end of the experiment. Mice subjected to GVHD and treated with vehicle developed the disease, which was confirmed by 100% lethality, 16 days after transplantation (Figure 1(a)), and high clinical scores (Figure 1(b)). In contrast, mice subjected to GVHD and treated with apocynin exhibited 83% survival (Figure 1(a)) and lower clinical scores over this period (Figure 1(b)). Treatment with apocynin was discontinued on day 16. On day 18, one mouse died, resulting in 67% overall survival until 30 days after transplantation, when the remaining mice were euthanized (Figure 1(a)). These findings are relevant because GVHD is still the main source of complication in allogeneic hematopoietic stem cell transplantation (allo-HSCT). Despite advances in the therapeutic strategies for the reduction in the incidence of this disease, about 35-50% of the transplanted patients develop grade II–IV acute GVHD [30], causing the death of 15-40% of these patients [1, 2, 8]. Therefore, treatment with apocynin may be exploited as a novel therapeutic strategy to treat GVHD.

To confirm the beneficial effects of apocynin treatment in GVHD, we tested it on mice that had been subjected to the allogeneic model of GVHD. Leukocytes isolated from C57BL/6 mice were transplanted into BALB/c mice, as described in Materials and Methods. Treatment with apocynin was also protective in this model (Supplementary [Supplementary-material supplementary-material-1]).

### 3.2. Apocynin Treatment Reduced Oxidative Stress in GVHD Target Organs

**Figure 2:**
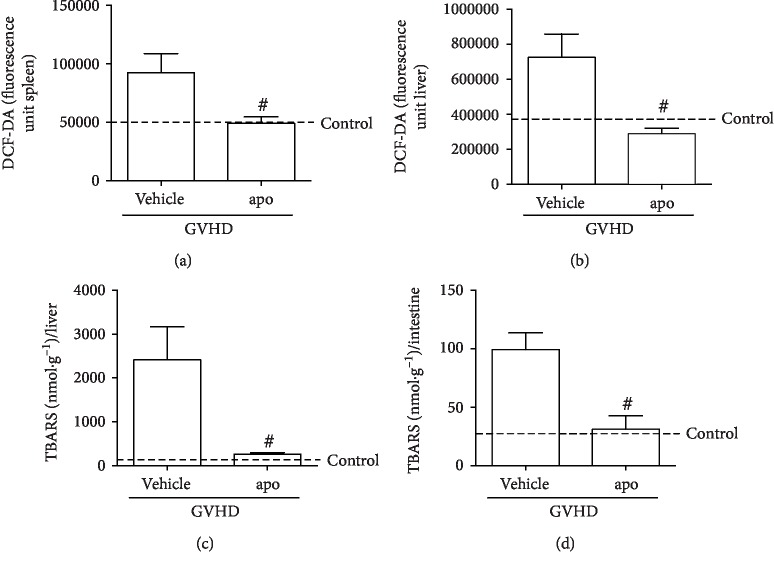
Apocynin treatment reduces reactive oxygen species and lipid peroxidation in GVHD target organs. GVHD was induced by the adoptive transfer of 10^7^ BM cells + 3 × 10^7^ splenocytes from C57BL/6 mouse donors to B6D2F1 mice. Mice that received syngeneic (B6D2F1) BM cells and splenocytes did not develop disease and were considered the control group. After GVHD induction, recipient mice were treated with apocynin (3 mg/kg, 24 h/24 h, intraperitoneally) or vehicle 30 min before transplantation until the experimental endpoint. At the onset of mortality, mice were killed, and the levels of reactive oxygen species were evaluated in the (a) spleen and (b) liver by DCF-DA analysis. The lipid peroxidation was also evaluated by TBARS in the (c) liver and (d) jejunum-ileum. Results are presented as the mean ± SEM (*n* = 4‐7); ∗ and #: *p* < 0.05 when comparing to the control and vehicle groups, respectively.

The level of ROS in the liver, spleen and bone marrow (BM) of mice with experimental GVHD was measured at day 13 after transplantation, which corresponded with the onset of mortality. There was an increase in ROS in the spleen and liver in the vehicle group, compared to that in the control group (Figures 2(a) and 2(b)). Pharmacological blockade of NADPH oxidase complex by apocynin reduced the level of ROS in these GVHD target organs (Figures 2(a) and 2(b)). The level of ROS in the BM was similar in mice treated with apocynin or vehicle (data not shown).

These results were supported by our recent work that demonstrated an increase in ROS production and liver injury in mice subjected to GVHD [13]. Amer and colleagues [31] have also demonstrated an increase in oxidative stress and ROS level in blood cells after allogeneic BM transplantation in mice. Moreover, several studies have shown that conditioning regimens result in the production of ROS in allo-HSCT patients, which contributes to the inflammatory response and tissue injury related to GVHD [32–34].

Next, we analyzed lipid peroxidation in the liver and jejunum-ileum using the thiobarbituric acid reactive substance (TBARS) assay. At day 13 after transplantation, there was an increase in TBARS levels in both organs, in mice subjected to GVHD and treated with vehicle. Treatment with apocynin inhibited lipid peroxidation related to GVHD in these organs (Figures 2(c) and 2(d)). High levels of ROS can cause oxidative stress, a phenomenon characterized by lipid peroxidation, protein destruction, cell death, and tissue injury [22, 35]. In this context, the reduction of ROS by apocynin treatment might have contributed to lower intestinal and hepatic damage and prevented GVHD-associated mortality and morbidity.

### 3.3. Treatment with Apocynin Reduced Hepatic and Intestinal Injuries Related to GVHD

**Figure 3:**
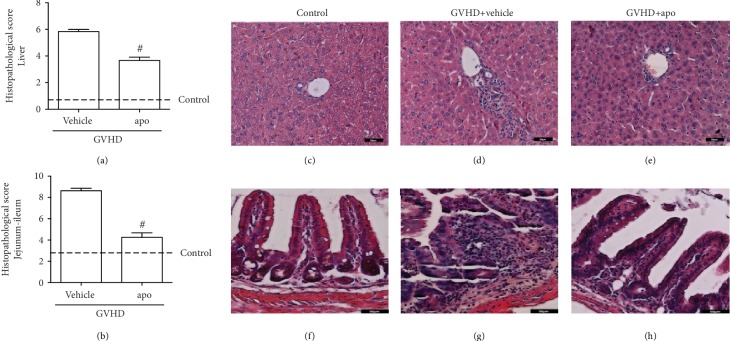
Apocynin treatment reduces hepatic and intestinal injuries related to GVHD. GVHD was induced by the adoptive transfer of 10^7^ BM cells + 3 × 10^7^ splenocytes from C57BL/6 mouse donors to B6D2F1 mice. Mice that received syngeneic (B6D2F1) BM cells and splenocytes did not develop disease and were considered the control group. After GVHD induction, recipient mice were treated with apocynin (3 mg/kg, 24 h/24 h, intraperitoneally) or vehicle 30 min before transplantation until the experimental endpoint. At the onset of mortality, the mice were killed, and the (a, c–e) liver and (b, f–h) jejunum-ileum tissues were sampled for histopathologic analysis. (c–e) Histological aspects of H&E-stained liver sections from the control, vehicle group, and apo group, respectively. (f–h) Histologic aspects of hematoxylin and eosin- (H&E-) stained small intestine sections from the control, vehicle group, and apo group, respectively. Scale bar: 50 *μ*m for all panels. Results are presented as the mean ± SEM (*n* = 4‐6); ∗ and #: *p* < 0.05 when comparing to the control and vehicle groups, respectively.

To confirm our hypothesis that the reduction in oxidative stress by apocynin contributes to less damage to GVHD target organs, we next evaluated the effect of apocynin treatment on the histopathologic alterations observed in the liver and intestine of mice subjected to GVHD. At day 13, the vehicle group presented severe liver injury throughout the parenchyma, including hepatocyte necrosis and diffused vacuolization. There was also increased inflammatory infiltration, mainly in the periportal areas (Figures 3(a) and 3(c–e)). In contrast, apocynin-treated mice showed substantial preservation of hepatic tissue and decreased inflammatory cell accumulation (Figures 3(a) and 3(c–e)). These findings are relevant because the liver is the second most frequent organ affected by GVHD, after skin [36]. About 80% of allo-HSCT patients develop hepatic aggravations, which contributes substantially to overall morbidity and mortality [37]. Hepatic GVHD is characterized by damage to bile duct epithelium, cytoplasmic eosinophilia, and vacuolation [38]. Injury to this organ is associated with clinical manifestation of the disease including hepatomegaly and icterus [39, 40]. Some studies have demonstrated that ROS can contribute to the death of hepatocytes, perpetuation of chronic inflammatory response, and the development of hepatic fibrinogenesis [41, 42]. We have previously shown that fullerol, a nanocomposite with antioxidant properties, also reduces hepatic injury and GVHD mortality, similar to apocynin treatment [13]. Our results corroborated with those obtained by Liu et al. [43], who have also demonstrated that the use of apocynin significantly decreased the production of ROS, leading to preservation of hepatic parenchyma, reduction in the cellular infiltrate, and limited necrosis in a murine model of hepatic ischemia and reperfusion.

Histological analysis of the jejunum-ileum of mice subjected to GVHD and treated with vehicle showed partial loss of organ architecture, hyperplasia of crypts, villous enlargement, edema, congestion, and increased cellularity. Severe degenerative changes, ulcerations of the mucosa, and areas of focal necrosis in the muscular and serous layers were also observed at this time point (Figures 3(b) and 3(f–h)). Apocynin treatment reduced the degenerative process in the intestine, resulting in the preservation of jejunum-ileum architecture and reduction in inflammatory infiltration into the lamina propria and in the muscle and serous layer (Figures 3(b) and 3(f–h)). About 50-60% of the patients receiving HSCT develop intestinal GVHD, which is associated with bacterial translocation, sepsis, and death [39, 44]. The loss of redox homeostasis characterized by high ROS levels and inhibition of antioxidant systems has been demonstrated to participate in the pathogenesis of many gastrointestinal diseases, such as Barrett's esophagus, esophageal adenocarcinoma, ischemic intestinal injury, celiac disease, inflammatory bowel disease, and colorectal cancer [45, 46]. Thus, we believe that the injury observed in GVHD target organs was produced in part by oxidative stress. Therefore, ROS inhibition and reduction in lipid peroxidation by apocynin may have contributed to reduced damage of the intestine and liver, improved survival, and decreased GVHD clinical score.

### 3.4. Inhibition of NADPH Oxidase Complex Reduced Proinflammatory Cytokines and Chemokines in GVHD Target Organs

**Figure 4:**
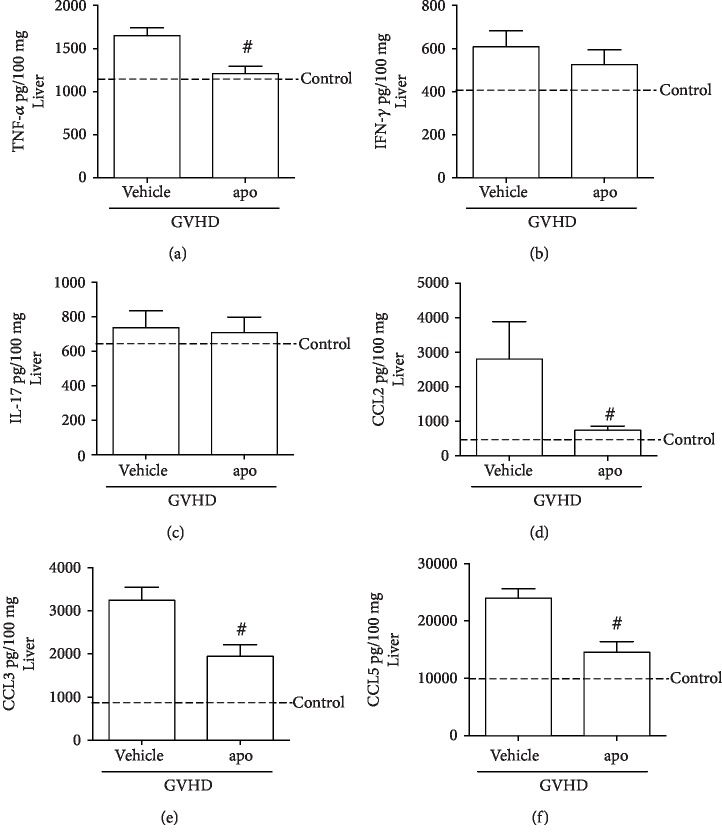
Apocynin treatment reduces the concentration of cytokines and chemokines in the liver of mice subjected to GVHD. GVHD was induced by the adoptive transfer of 10^7^ BM cells + 3 × 10^7^ splenocytes from C57BL/6 mouse donors to B6D2F1 mice. Mice that received syngeneic (B6D2F1) BM cells and splenocytes did not develop disease and were considered the control group. After GVHD induction, recipient mice were treated with apocynin (3 mg/kg, 24 h/24 h, intraperitoneally) or vehicle 30 min before transplantation until the experimental endpoint. At the onset of mortality, mice were killed, and the concentrations of (a) TNF, (b) IFN-*γ*, (c) IL-17, (d) CCL2, (e) CCL3, and (f) CCL5 in the hepatic homogenates were evaluated by ELISA. Results are presented as the mean ± SEM (*n* = 6‐10); ∗ and #: *p* < 0.05 when comparing to the control and vehicle groups, respectively.

**Figure 5:**
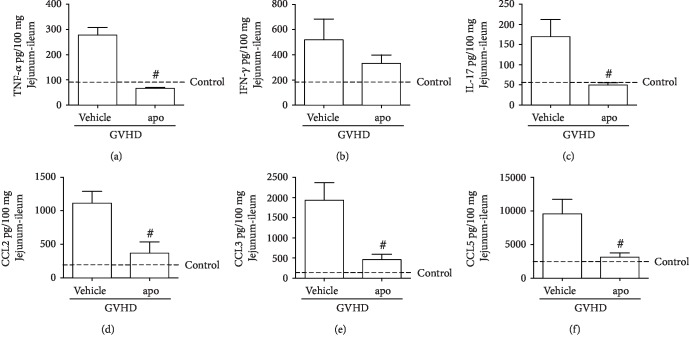
Apocynin treatment reduces the concentration of cytokines and chemokines in the jejunum-ileum of mice subjected to GVHD. GVHD was induced by the adoptive transfer of 10^7^ BM cells + 3 × 10^7^ splenocytes from C57BL/6 mouse donors to B6D2F1 mice. Mice that received syngeneic (B6D2F1) BM cells and splenocytes did not develop disease and were considered the control group. After GVHD induction, recipient mice were treated with apocynin (3 mg/kg, 24 h/24 h, intraperitoneally) or vehicle 30 min before transplantation until the experimental endpoint. At the onset of mortality, mice were killed, and the concentrations of (a) TNF, (b) IFN-*γ*, (c) IL-17, (d) CCL2, (e) CCL3, and (f) CCL5 in the intestinal homogenates were evaluated by ELISA. Results are presented as the mean ± SEM (*n* = 6‐10); ∗ and #: *p* < 0.05 when comparing to the control and vehicle groups, respectively.

The levels of TNF*α*, IFN*γ*, IL-17, CCL2, CCL3, and CCL5 were increased in the liver and jejunum-ileum of mice subjected to GVHD and treated with vehicle at 13 days after transplantation (Figures 4 and 5). In accordance with reduced overall hepatic and intestinal histopathological score, the main inflammatory mediators that orchestrate GVHD target organ injury were decreased by apocynin treatment, including TNF*α*, CCL2, CCL3, and CCL5 in the liver (Figures 4(a) and 4(d–f)) and TNF*α*, IL-17, CCL2, CCL3, and CCL5 in the small intestine (Figures 5(a), 5(c), and 5(d–f)).

Previous studies by our group have explored the participation of these proinflammatory molecules in GVHD pathophysiology [12–16, 25]. The role of TNF*α* in GVHD development has been supported by several clinical trials which have demonstrated a strong correlation between increased levels of this cytokine and GVHD. A study of 61 GVHD patients treated with corticosteroids and etanercept, a TNF*α* inhibitor, found significant improvement in the disease symptoms compared to patients treated with corticosteroids alone [47]. Furthermore, TNF participates in the activation of antigen-presenting cells (APC) and stimulates mononuclear cell recruitment and proliferation of cytotoxic T cells [6]. Interestingly, Scott et al. [48] showed that ROS regulates the activity of TNF*α*-converting enzyme (TACE), which is responsible for the cleavage of TNF from the membrane to its soluble form. TNF*α* also plays a role in enhancing the activity of NADPH oxidase in macrophages, monocytes, and neutrophils, inducing ROS production [49]. Thus, ROS inhibition by apocynin after GVHD induction may be related to reduced levels of TNF*α*, which in turn may also contribute to the further reduction of ROS, accentuating the decrease in the levels of this mediator. Apocynin treatment also reduced the levels of TNF*α* in a model of hepatic ischemia and reperfusion in mice, thereby corroborating our results [43].

The reduction of IL-17 in the intestine after apocynin treatment is also relevant because this cytokine can be produced by CD4^+^ and CD8^+^ T cells and is related to tissue inflammation in GVHD target organs [12, 50]. Kappel and colleagues [51] have shown that the transplantation of murine IL-17(-/-) CD4(+) T cells delayed GVHD development. A recent study by He et al. [52] also showed that increased production of ROS by peritoneal macrophages promoted the elevation of IL-17 levels in peritoneal lavage and subsequently intensified the inflammatory response in a sepsis model in mice. Thus, NADPH oxidase inhibition mediated by apocynin may also contribute to the reduction in intestinal levels of IL-17 as shown here.

Multiple studies have shown the participation of the chemokines CCL2, CCL3, and CCL5 in GVHD [12–16, 25, 53–56]. In our previous study, we found that the absence of CCL3 in donor leukocytes or the pharmacological blockade of its receptor also prevents mortality and reduces damage in the intestine and liver of mice subjected to GVHD [16]. Furthermore, CCL2 and CCL5 are related to recruitment of lymphocytes and macrophages to target organs of GVHD, perpetuating an exacerbated immune response [15, 53–55]. Corroborating our results of chemokine reduction by apocynin, some studies have been demonstrated that this NADPH oxidase inhibitor also reduces the levels of CCL2 and CCL5 in other experimental models, such as influenza A virus infection [57] and atherosclerosis [58].

Together, our data highlights the ability of apocynin to reduce proinflammatory cytokines and chemokines and consequently, to alleviate GVHD.

### 3.5. Apocynin Treatment Reduces Inflammatory Infiltrate in GVHD Target Organs

**Figure 6:**
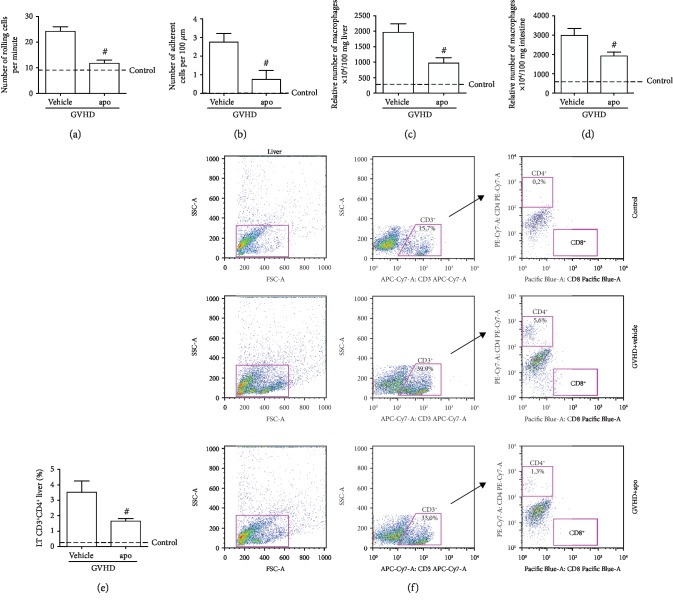
Apocynin treatment reduces leukocyte recruitment into GVHD target organs. GVHD was induced by the adoptive transfer of 10^7^ BM cells + 3 × 10^7^ splenocytes from C57BL/6 mouse donors to B6D2F1 mice. Mice that received syngeneic (B6D2F1) BM cells and splenocytes did not develop disease and were considered the control group. After GVHD induction, recipient mice were treated with apocynin (3 mg/kg, 24 h/24 h, intraperitoneally) or vehicle 30 min before transplantation until the experimental endpoint. The leukocyte recruitment was evaluated on day 13 after transplantation. The mice were anesthetized, and intestinal venules (±40 *μ*m) were selected to count the numbers of rolling and adherent leukocytes by intravital microscopy. (a) The number of rolling cells/minute; (b) the number of adherent cells/100 *μ*m. Results are presented as the mean ± SEM (*n* = 4). Macrophages were quantified in the (c) liver and (d) jejunum-ileum by enzymatic methods (NAG assay). Results are presented as the mean ± SEM (*n* = 6‐9). The percentage of (e) hepatic LT CD4^+^ was evaluated by flow cytometry. Results are presented as the mean ± SEM (*n* = 4). (f) Representative dot plot of the flow cytometry analysis. ∗ and #: *p* < 0.05 when comparing to the control and vehicle groups, respectively.

To assess whether apocynin could interfere with the initial stages of leukocyte migration to the GVHD target organs, we performed intravital microscopy on the intestinal postcapillary venules of animals subjected to GVHD, at day 13 after transplantation. Treatment with apocynin reduced the numbers of both rolling and mesenteric venule-adherent leukocytes (Figures 6(a) and 6(b)). This result was similar to the findings published by Nunes-Silva et al. [59], who demonstrated the relevance of apocynin in decreasing the expression of adhesion molecules and subsequently, inhibiting cell recruitment into muscle tissue.

Moreover, apocynin treatment reduced the accumulation of macrophages in the liver and jejunum-ileum (Figures 6(c) and 6(d)) and the frequency of CD4^+^ T cells in the liver (Figures 6(e) and 6(f)). There was no difference in the frequency of CD4^+^ T cells in the jejunum-ileum and in the frequency of CD8^+^ T cells in the liver and jejunum-ileum of mice treated with apocynin or vehicle (data not shown). The increase in ROS concentration leads to a differential T cell response, including T cell receptor (TCR) activation and cytokine production. On the other hand, the reduction in ROS levels leads to low T cell activation and proliferation [60]. The activation and interaction between donor T cells and host APCs are essential for GVHD development. The interference in T lymphocyte function or its depletion through pharmacological and experimental manipulation is a common strategy for GVHD prophylaxis [2]. Tissue damage caused by cytotoxic T lymphocytes induces the recruitment of other effector cells including natural killer (NK) cells, neutrophils, and macrophages. Macrophages also have an important role as APCs in the efferent phase of acute GVHD. The progressive activation or priming of these cells results in production of inflammatory mediators such as TNF*α* and ROS, which causes subsequent inflammatory tissue injury, weight loss, and death [61, 62]. This mechanism of reduction in inflammatory infiltrate in the intestine and liver after apocynin treatment observed here is consistent with our previous results showing less injury and lower levels of proinflammatory cytokines and chemokines in GVHD target organs after ROS inhibition.

### 3.6. Inhibition of NADPH Oxidase Complex Did Not Interfere with Chimerism

**Figure 7:**
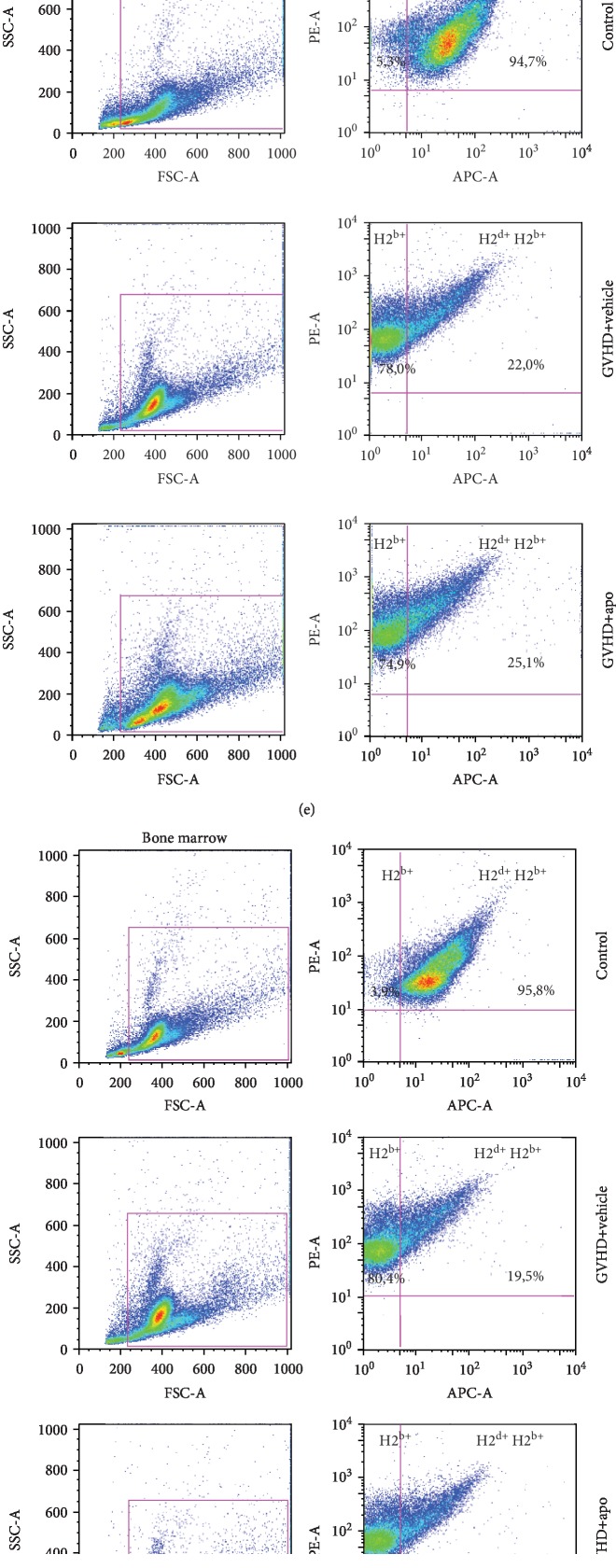
Apocynin treatment did not interfere with chimerism. GVHD was induced by the adoptive transfer of 10^7^ BM cells + 3 × 10^7^ splenocytes from C57BL/6 mouse donors to B6D2F1 mice. Mice that received syngeneic (B6D2F1) BM cells and splenocytes did not develop disease and were considered the control group. After GVHD induction, recipient mice were treated with apocynin (3 mg/kg, 24 h/24 h, intraperitoneally) or vehicle 30 min before transplantation until the experimental endpoint. At day 13 after transplant, the mice were killed and the percentage of H2^d+^H2^b+^ cells (marker for B6D2F1 cells) and H2^b+^ cells (marker for C57BL/6 cells) in the spleen and BM was evaluated by flow cytometry. (a) Frequency of H2^b+^ in the spleen. (b) Frequency of H2^b+^H2^d+^ in the spleen. (c) Frequency of H2^b+^ in the BM. (d) Frequency of H2^b+^H2^d+^ in the BM. (e) Representative dot plot of the flow cytometry analysis in the spleen. (f) Representative dot plot of the flow cytometry analysis in the BM. Results are presented as the mean ± SEM (*n* = 4); ∗ and #: *p* < 0.05 when comparing to the control and vehicle groups, respectively.

Stable donor engraftment is essential for the reconstitution of the hematopoietic niche of transplanted patients and for the elimination of reminiscent tumor cells after hematopoietic stem cell transplant [63]. The main goal of allo-HSCT is complete donor hematopoietic chimerism. The maintenance of receptor hematopoiesis is related to the risk of relapse and mortality after transplantation [64]. Our next step was to evaluate if apocynin treatment could interfere with engraftment. In order to achieve this objective, we assessed the frequency of H2^d+^H2^b+^ cells (a marker of B6D2F1 cells) and H2^b+^ cells (a marker of C57BL/6 cells) in the spleen and BM at day 10 after transplantation. The control group presented mostly H2^d+^H2^b+^ cells in both the spleen and BM (Figure 7). Apocynin or vehicle-treated mice showed a high frequency of H2^b+^ cells and a low frequency of H2^d+^H2^b+^ cells in both spleen and BM (Figure 7). These results indicated that apocynin treatment did not interfere with chimerism, confirming the success of engraftment.

## 4. Conclusions

**Figure 8:**
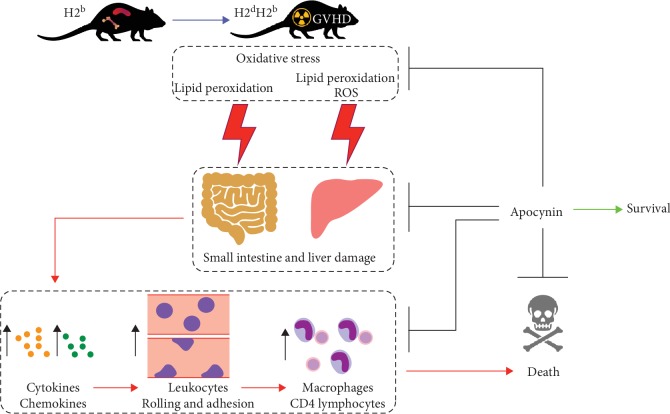
Summary of GVHD protection induced by apocynin treatment. Apocynin treatment controls oxidative stress, which in turn results in lower liver and intestinal damage, decreased levels of proinflammatory cytokines and chemokines, reduced leukocyte rolling and adhesion, reduced recruitment of macrophages and CD4 lymphocytes to GVHD target organs, and improvement of survival.

Altogether, our data indicate that apocynin could be a potential therapeutic effect against acute GVHD. The ability of this NADPH oxidase inhibitor to increase survival and reduce severity of acute GVHD appears to be associated with controlling oxidative stress, which in turn results in lower liver and intestinal damage, reduced levels of proinflammatory cytokines and chemokines, and decreased leukocyte recruitment to GVHD target organs (Figure 8). Importantly, apocynin was able to regulate the inflammatory response related to GVHD without impairing the engraftment.

## Data Availability

The graph data used to support the findings of this study are included within the article and within the supplementary information file.

## Supplementary Materials

Supplementary Materials Supplementary Figure 1: apocynin treatment reduces lethality and clinical signs in an allogeneic model of GVHD.
