# Supplementary material for: Treatment with Apocynin Limits the Development of Acute Graft-versus-Host Disease in Mice
Source: J Immunol Res. 2019 Nov 3;2019:9015292. doi: 10.1155/2019/9015292 (PMC6874984; doi:10.1155/2019/9015292)
Supplement: Supplementary Materials — Supplementary Figure 1: apocynin treatment reduces lethality and clinical signs in an allogeneic model of GVHD. [file 9015292.f1.pdf]

Supplementary Materials

Supplementary figure 1

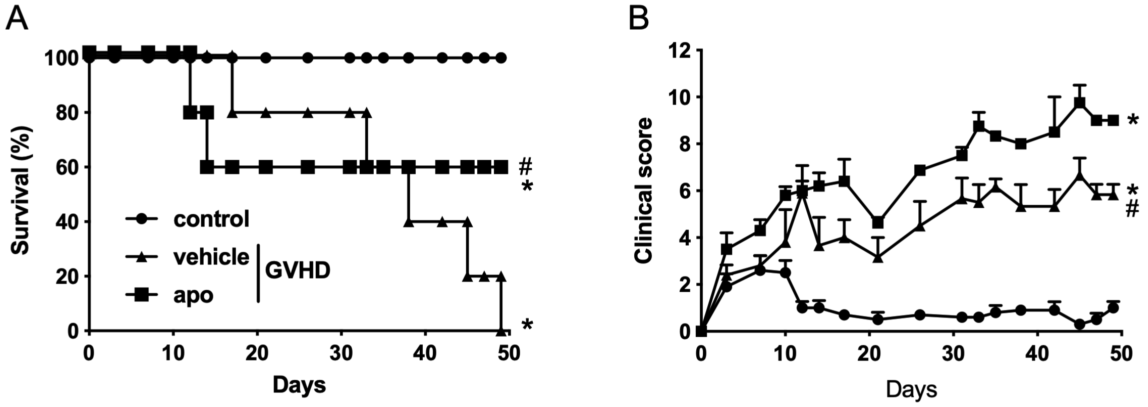

Supplementary figure 1. Apocynin treatment reduces lethality and clinical signs in an allogeneic model of GVHD. GVHD was induced by the adoptive transfer of 10^7 BM cells + 10^7 splenocytes from C57BL/6 mice donors to BALB/c mice. Mice that received syngeneic (BALB/c) BM cells and splenocytes did not develop disease and were considered the control group. After GVHD induction, recipient mice were treated with apocynin (3 mg/kg, 24 h/24 h, intraperitoneally) or vehicle 30 min before transplantation until the experimental endpoint. The mice were evaluated every 2 d for survival (A) and clinical scoring (B). The results are shown as means ± SEM, and the numbers of animals were as it follows: control group (●, n=5); vehicle group (▲, n=6); apo group (■, n=6). * and # $P < 0.05$ compared with the control and vehicle groups, respectively.
